# Effects of PSA Removal from NCAM on the Critical Period Plasticity Triggered by the Antidepressant Fluoxetine in the Visual Cortex

**DOI:** 10.3389/fncel.2016.00022

**Published:** 2016-02-05

**Authors:** Ramon Guirado, Danilo La Terra, Mathieu Bourguignon, Hector Carceller, Juzoh Umemori, Pia Sipilä, Juan Nacher, Eero Castrén

**Affiliations:** ^1^Neuroscience Center, University of HelsinkiHelsinki, Finland; ^2^Department of Neuroscience and Biomedical Engineering, Aalto UniversityHelsinki, Finland; ^3^Basque Center on Cognition, Brain and LanguageDonostia, Spain; ^4^Departamento de Biologia Celular, Spanish National Network for Research in Mental Health, CIBERSAM, Fundación Investigación Hospital Clínico de Valencia, INCLIVA, Universitat de ValenciaValencia, Spain; ^5^Max Planck Institute for NeurobiologyMartinsried, Germany

**Keywords:** fluoxetine, critical period plasticity, PSA-NCAM, parvalbumin interneurons, visual plasticity

## Abstract

Neuronal plasticity peaks during critical periods of postnatal development and is reduced towards adulthood. Recent data suggests that windows of juvenile-like plasticity can be triggered in the adult brain by antidepressant drugs such as Fluoxetine. Although the exact mechanisms of how Fluoxetine promotes such plasticity remains unknown, several studies indicate that inhibitory circuits play an important role. The polysialylated form of the neural cell adhesion molecules (PSA-NCAM) has been suggested to mediate the effects of Fluoxetine and it is expressed in the adult brain by mature interneurons. Moreover, the enzymatic removal of PSA by neuroaminidase-N not only affects the structure of interneurons but also has been shown to play a role in the onset of critical periods during development. We have here used ocular dominance plasticity in the mouse visual cortex as a model to investigate whether removal of PSA might influence the Fluoxetine-induced plasticity. We demonstrate that PSA removal in the adult visual cortex alters neither the baseline ocular dominance, nor the fluoxetine-induced shift in the ocular dominance. We also show that both chronic Fluoxetine treatment and PSA removal independently increase the basal FosB expression in parvalbumin (PV) interneurons in the primary visual cortex. Therefore, our data suggest that although PSA-NCAM regulates inhibitory circuitry, it is not required for the reactivation of juvenile-like plasticity triggered by Fluoxetine.

## Introduction

Critical periods during development have been characterized as time-windows of high plasticity during which the external milieu shapes networks and behavior (Katz and Shatz, 1996; Berardi et al., 2000). In the visual cortex, the classical studies of Hubel and Wiesel showed how ocular dominance columns are established during a critical period in postnatal development of the visual system in cats (Wiesel and Hubel, 1963). After that time-window of enhanced plasticity, the established ocular dominance will remain permanent (Hubel and Wiesel, 1970). Disturbance of normal binocular vision during such critical period, in animal models usually by monocular deprivation, leads to disrupted integration of visual information in the cortex. The altered balance of information arriving to the visual cortex from the occluded eye results in reduced visual acuity and contrast sensitivity (Dews and Wiesel, 1970; Fagiolini et al., 1994; Prusky et al., 2000), a condition known in humans as amblyopia (Webber and Wood, 2005).

Although the visual system of rodents lacks characteristic columns described in higher mammals, critical periods for ocular dominance have nevertheless been extensively characterized in rodents as well (Kirkwood and Bear, 1994; Kirkwood et al., 1995; Huang et al., 1999). Interestingly, recent reports have shown that a critical period-like plasticity can be reopened in the adult visual cortex through different experimental manipulations (Sale et al., 2009; Bavelier et al., 2010; Beurdeley et al., 2012) including antidepressant drugs such as Fluoxetine (Maya-Vetencourt et al., 2008; Tiraboschi et al., 2013). The combination of Fluoxetine with monocular deprivation of one eye in adult rats produces a shift in the ocular dominance towards the open eye. This shift resembles that achieved by the same paradigm of monocular deprivation during the developmental critical period (Maya-Vetencourt et al., 2008).

Cellular and molecular mechanisms that have been connected with the Fluoxetine-induced opening of critical periods in adult brain include rearrangement of inhibitory circuits (Maya-Vetencourt et al., 2008), signaling by Brain-derived neurotrophic factor (BDNF; Maya-Vetencourt et al., 2008), serotoninergic transmission (Maya-Vetencourt et al., 2010; Ferrés-Coy et al., 2015) and the transcription factor NPAS4 (Maya-Vetencourt et al., 2012), but detailed molecular mechanisms through which Fluoxetine reopens critical periods are still unclear (Maya-Vetencourt et al., 2012; Sharp, 2013; Tiraboschi et al., 2013). A promising candidate to mediate this mechanism is the polysialylated form of neural cell adhesion molecules (PSA-NCAM), a molecule widely expressed during development but subsequently restricted, in the adult neocortex, to a subpopulation of interneurons (Sandi, 2004; Rutishauser, 2008). PSA-NCAM is a negatively charged hydrophilic molecule that creates a steric impediment affecting membrane receptors and signaling (Burgess and Aubert, 2006; Gascon et al., 2007). NCAM is the major polysialylated molecule in the adult brain and its polysialylation has been linked with adult brain plasticity (Bonfanti, 2006; Nacher et al., 2013). Particularly, chronic antidepressant treatments have been reported to increase PSA-NCAM expression in different brain regions (Sairanen et al., 2007; Varea et al., 2007; Guirado et al., 2014b). Conversely, enzymatic removal of PSA has been shown to disrupt plastic mechanisms such as LTP (Muller et al., 1996; Dityatev et al., 2004); effects that can be counteracted by BDNF supplementation (Muller et al., 2000). In addition, PSA removal has also been suggested to trigger an earlier onset of critical periods in the visual cortex through the precocious maturation of the perisomatic innervation of pyramidal neurons by basket cells and enhancing inhibitory synaptic transmission (Di Cristo et al., 2007). In fact, PSA depletion during adulthood also induces an increase in this perisomatic inhibitory input (Castillo-Gómez et al., 2011). All these studies suggest that regulation of PSA-NCAM expression is a possible mechanism mediating the effects of Fluoxetine in the adult brain plasticity.

To investigate the interaction between PSA-NCAM and Fluoxetine, we studied the effects of enzymatic removal of the polysialic acid moiety of PSA-NCAM on a critical period plasticity paradigm: the shifts of ocular dominance in the visual cortex during Fluoxetine chronic treatment.

## Materials and Methods

### Animals

Forty-eight C57BL/6 female mice (Harlan Laboratories, Netherlands) were used. Animals were divided into 4 different groups of 12 to control two experimental manipulations: Fluoxetine treatment and Endoneuraminidase-N (endoN) intracerebral injections (group 1: no manipulation; group 2: Fluoxetine only; group 3: EndoN only; group 4: Fluoxetine and EndoN). The animals were caged in groups of four and kept under standard housing conditions (21°C, 12 h light/12 h dark cycle, lights on at 6 AM). Mice were 3 months old at the beginning of the experiment, ensuring that the shift in the ocular dominance took place after P110 when the extended critical period has been suggested to end (Greifzu et al., 2014). All animals had free access to food and water for the entire duration of the experimentation. All procedures were carried out according to the Directive 2010/63/EU of the European Parliament and were approved by the Animal Ethical Committee of Southern Finland.

### Visual Abilities

Following a previously described methodology (Drapeau et al., 2014), general visual acuity was tested by using the visual placing test, which takes advantage of the forepaw-reaching reflex: the mouse was held by its tail approximately 20 cm above the surface and was then progressively lowered. As it approaches the surface, the mouse should expand its forepaws to reach the floor. The test was repeated three times with a 30 s interval, and the distance from the ground when the animals show the forepaw reflex without using their whiskers was quantified.

### Fluoxetine Treatment

**Figure 1:**
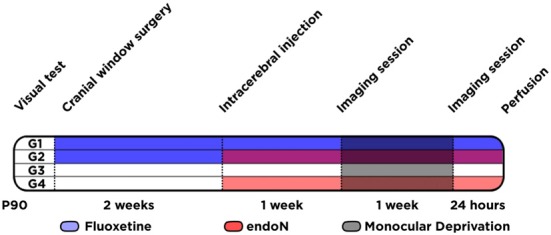
**Experimental design.** Schematic graph showing the different animal groups (G1 to G4) and the different experimental conditions conducted at different time-points.

Mice received Fluoxetine via drinking water. Drinking water was supplemented with 0.16 mg/mL Fluoxetine and 0.5% sucrose. Solutions were prepared fresh twice a week, and drinking bottles protected from light. We started the treatment immediately after the cranial window surgery, and it lasted for 4 weeks until the end of the experiment (Figure 1). The level of Fluoxetine/water consumed and animal bodyweight were constantly monitored to evaluate drug dose, which was approximately 20 mg/Kg/day. This protocol of chronic Fluoxetine administration to adult mice was reported to result in clinical relevant plasma levels of the drug (Dulawa et al., 2004).

### Chronic Cranial Window Surgical Procedure

Three weeks prior to the first imaging session animals were implanted flush with cranial windows in their right hemisphere, following a protocol adapted from Holtmaat et al. (2009). In short: anesthesia was induced with 3–4% Isoflurane (Vetflurane), then continued with intraperitoneal (IP) injection of sleeping cocktail: Fentanyl (opioid receptor agonist; 0.05 mg/kg), Midazolam (GABA-A agonist; 5.0 mg/Kg) and Medetomidine (adrenergic receptor agonist; 0.5 mg/Kg). Animals were placed in a stereotaxic frame, the skin and periosteum were removed and the right temporalis muscles separated from the bone. With this procedure a large area of the skull was exposed. Using a small amount of cyanoacrylate (Vetbond) we seal the skin on the sides. A circular groove was drawn using a surgical drill. Then, the bone piece was lifted leaving the dura intact. A 5 mm diameter circular cover glass (EMS) was flushed on. Glue gel (Loctite) was applied around the borders and under a metal head bar to glue it to the skull. Finally, dentist cement was applied around the craniotomy to cover all tissues exposed, except a small area ipsilateral to the cranial window, covered with putty for the following intracerebral injection.

### Stereotaxic Injection of EndoN

As previously described, mice were anesthetized first with isoflurane and then with a sleeping cocktail. Additionally, animals were injected subcutaneously with carprofen (Rimadyl; 5 mg/Kg) and intramuscularly with dexamethasone (0.6 mg/Kg), for pre-emptive analgesia and anti-inflammatory effects. Then placed in a stereotaxic frame, the piece of putty was removed for the intracerebral injection. The enzyme endoN (0.7 U/μL in glycerol; AbCys) or the vehicle solution (1 μl; saline and glycerol 1:1) was injected using a Hamilton syringe in the ipsilateral hemisphere to the cranial window within the somatosensory cortex (antero-posterior −0.5 mm, medio-lateral −1.5 mm, dorso-ventral −0.7 mm, relative to Bregma). The needle was left in position for 1 min and then 1 μL of endoN or vehicle was injected during another minute. After the injection was completed, the needle was left in place for 3 min to reduce reflux of the solution into the track of the injection needle and then slowly withdrawn.

After all surgical procedures, animals received an IP injection with a wake-up cocktail containing antagonists of the same receptors affected by the sleeping cocktail: Naloxone (1.2 mg/Kg), Flumazenil (0.5 mg/Kg) and Atipamezole (2.5 mg/Kg).

### *In Vivo* Imaging of Intrinsic Signals

We adapted our methodology from a previous protocol described by the laboratory of Dr Stryker (Kalatsky and Stryker, 2003; Cang et al., 2005) to measure saturation of hemeoglobin as an indicator of cortical activity in the mouse visual cortex. Mice were injected IP with a lower dose (2/3 of the surgical dose) of the sleeping cocktail to produce a shallow anesthesia. Then, animals were placed in the setup, under a high frequency CCD camera (Brain Imager 3001/S) 25 cm in front of a screen. The cranial window was illuminated with red light using a broad pass filter (630 ± 20 nm), and the camera focused 500 μm below the surface area with a field of view roughly matching the full extent of the cranial window. We produced a visual stimulus consisting in a two degrees-thick horizontal bar in a position between −15 and +5 degrees azimuth (relative to the field of vision of the animal). The bar flickered at 6 Hz and drifted vertically with cycles corresponding to 0.11 Hz to elicit rhythmic activity at the same frequency in the right hemisphere from both ipsilateral and contralateral eyes. Four stimulation protocols were performed: two for each eye, and within one eye one moving upwards and one downwards. In each protocol, images of dimensions 336 × 336 pixels were sampled at 10 Hz for 5 min.

### Monocular Deprivation

After the first imaging session animals still under light anesthesia were implanted with eye occluders in the left eye (contralateral to the cranial window). These occluders were made as previously described (Park et al., 2005). In short, they were made from 0.25 mm thick black vinyl sheets that were heated and molded over a steel ball similar in size to the mice eyes. Mice from all groups were subjected to monocular deprivation during 7 days, after which they underwent a second imaging session. In a separate pilot experiment we ensured that there are no responses in the visual cortex when the animals wearing occluders are exposed to the same visual stimulus.

### Analysis of Imaging Data

Images were analyzed offline with custom-made scripts in matlab (MATLAB 7.8, The MathWorks, Inc). These scripts are freely available upon request. Images were stacked in the time-domain and Fourier-transformed in that dimension to extract a map of signal amplitude and phase at 0.11 Hz (the stimulation frequency). Amplitude images for stimuli moving upwards and downwards were averaged together, leaving us with one amplitude image per eye (contralateral and ipsilateral). Images were further smoothed with a 10-pixels-wide full-width-at-half-maximum Gaussian kernel in order to decrease the noise level. A region of interest that in principle matches with the location of the primary visual cortex was then defined as the pixels for which the image for the contralateral eye take values above 70% of the maximum of that same image. Finally, the ocular dominance index (ODI) was defined as *ODI* = (*A*_contra_ − *A*_ipsi_)/(*A*_contra_ + *A*_ipsi_), where *A*_contra_ (and *A*_ipsi_ respectively) is the mean amplitude of the image for the contralateral (resp. ipsilateral) eye within the region of interest. Phase maps masked outside the region of interest were used to confirm that cortical activity is entrained according to the known retinotopy; i.e., with a phase in each cortical areas directly related to its most responsive polar angle. Mice who failed to demonstrate systematic activity at 0.11 Hz and expected retinotopy were excluded from further analysis of the ODI.

### Perfusion

One day after the second *in vivo* imaging session, animals were perfused transcardially under deep pentobarbital anesthesia, first with saline and then with PFA 4% in PB 0.1 M. After perfusions, brains were extracted and postfixed in the same fixative solution for 2 h, then stored in PB 0.1 M with sodium azide 0.05%. Then brains were cut into 50 μm sections using a vibratome (VT 1000E, Leica). Sections were collected in six different subseries and stored at 4°C in PB 0.1 M and sodium azide.

#### Immunohistochemistry

Tissue was processed free-floating for fluorescence immunohistochemistry as follows. Sections were washed in PBS, then slices were incubated in 10% normal donkey serum (NDS; Gibco), 0.2% Triton-X100 (Sigma) in PBS for 1 h. Sections were then incubated for 48 h at 4°C with IgM mouse anti-PSA-NCAM (1:500; DSHB) or with a cocktail containing IgG mouse anti-parvalbumin (1:2000; Swant) and IgG rabbit anti-FosB (1:500; Santa Cruz) antibodies diluted in PBS 0.2% Triton-X100. After washing again, sections were incubated for 2 h at room temperature with secondary antibody anti-mouse IgM conjugated with A488 (1:200; Life Technologies) or with a cocktail containing: anti-mouse IgG conjugated with Alexa 647 and anti-rabbit IgG conjugated with Alexa 488 (1:200; Life Technologies). Sections were then washed in PB 0.1 M, mounted on slides and coverslipped using fluorescence mounting medium (Dako).

#### Confocal Imaging

Sections were analyzed using a confocal microscope (Zeiss LSM 700). We evaluated PSA-NCAM expression and quantified PV and FosB fluorescence intensity in two different sections containing primary visual cortex (between +3 and +3.7 mm in the Antero-Posterior relative to Bregma). Stacks were obtained at an optimal penetration depth in the tissue (1–6 μm deep). We used same acquiring conditions (as any other previous condition) to compare then the intensity of fluorescence between the samples. For PSA-NCAM quantification, small square areas of 500 μm^2^ were analyzed in deep layers of two different sections within the primary visual cortex of each animal. For FosB and PV quantification, all PV neurons were counted in that same optimal penetration depth in the tissue, then the nucleus stained with FosB was profiled and its fluorescence intensity quantified for both channels: FosB and PV. A total of 639 PV neurons were analyzed, although for statistical purposes we always used the number of animals as “*n*”. All animals were coded and the code was not broken until the end of the study.

## Results

### EndoN Efficiently Depletes PSA-NCAM in the Visual Cortex

**Figure 2:**
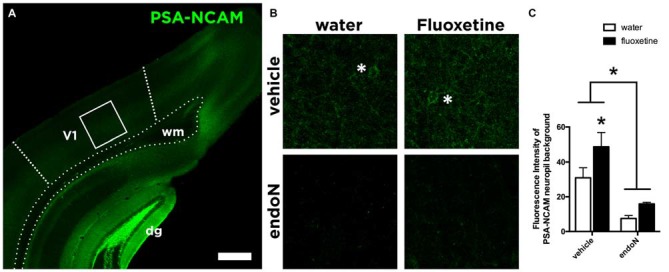
**PSA-NCAM expression in the Visual Cortex. (A)** Confocal single plane showing the expression of PSA-NCAM in the primary visual cortex of control mice. **(B)** Confocal single planes showing the expression of PSA-NCAM under different experimental conditions. Asterisks indicate the presence of PSA-NCAM positive interneurons in the animals injected with vehicle, while they are absent in the animals injected intracerebrally with endoN. **(C)** Graphs showing the expression of PSA-NCAM in our experimental conditions. EndoN removes effectively PSA-NCAM (2-way ANOVA), while in animals injected intracerebrally with vehicle, Fluoxetine increases the expression of PSA-NCAM (Fisher’s LSD) Mean ± SEM (water-vehicle = 30.92 ± 5.8, water-endoN = 7.5 ± 1.7, Fluoxetine-vehicle = 48.75 ± 8.1, Fluoxetine-endoN = 15.88 ± 0.95) *n* = 28 and *p < 0.05. Scale bar = 400 μm in **(A)** and 43 μm in **(B)**.

We analyzed the expression of the PSA-NCAM in the primary visual cortex of all the animals (Figure 2A). Intracerebral injections of the enzyme endoN produced a clear depletion of PSA-NCAM. Two weeks after the injection of the enzyme, the depletion covered most parts of the telencephalon, including always the visual cortex of the right hemisphere where the cranial windows were placed (Figure 2B). In these areas the enzyme effectively removed PSA-NCAM (2-way ANOVA; endoN effect: *F*_(1,24)_ = 17.02 and *p* = 0.0004; Figure 2C). In addition, Fluoxetine treatment increased the expression of PSA-NCAM (Fisher’s LSD; *p* = 0.0004) in control animals as previously described (Varea et al., 2007).

### Fluoxetine Produces a Shift in the Ocular Dominance but EndoN Does Not

**Figure 3:**
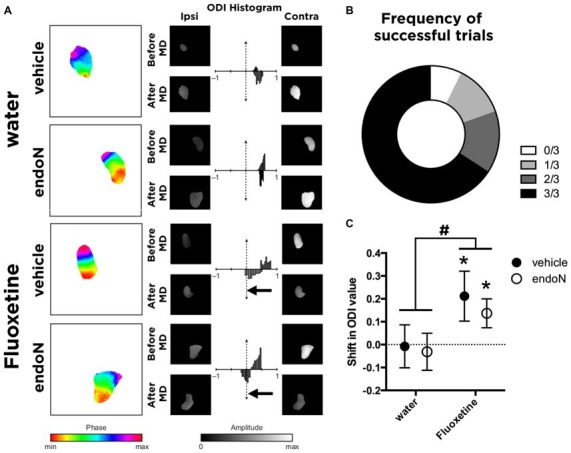
***In vivo* imaging of intrinsic signals. (A)** Color-coded phase maps and gray-coded amplitude maps of activity evoked by the visual stimulus in a representative animal from each group. Amplitude maps are displayed with a threshold at 70% of their maximal value, and the same masking was then applied to amplitude maps. Histograms represent the ocular dominance index (ODI) value (by comparing the magnitude in response between the ipsilateral and contralateral eye stimulation) before monocular deprivation (MD; upper part of each histogram) and after MD (lower part of each histogram). The arrows indicate significant shifts in the ocular dominance. **(B)** Graph showing the frequency distribution of successful trials in the forepaw-reflex behavioral test. **(C)** Graph showing the mean ± SEM of the shift in the ODI values (before and after MD; paired *t*-test; *p < 0.05). ^#^Indicates that Fluoxetine as a factor produces a shift in the ODI compared to water-drinking groups (2-way ANOVA; Mean ± SEM: water-vehicle = −0.0075 ± 0.093, water-endoN = −0.03125 ± 0.081, Fluoxetine-vehicle = 0.2117 ± 0.109, Fluoxetine-endoN = 0.1367 ± 0.063 ^#^*p* < 0.05; for more details, see “Results” Section).

Before the start of the experiment, all animals were evaluated for gross visual acuity. Three animals failed to show the forepaw reflex without using their whiskers and were removed from the study. The rest of the animals, showing at least one successful forepaw reflex without using the whiskers, were therefore used for the paradigm of visual plasticity (Figure 3B).

We then analyzed the effects of Fluoxetine and endoN in a paradigm of visual plasticity. By measuring the activity during visual stimulation in each eye, we calculated values of ODI as previously described (Cang et al., 2005) for each animal before and after monocular deprivation (Figure 3A). Briefly, we calculated the shift in ODI for each animal that occurred between the first imaging session (before monocular deprivation) and the second imaging session (after monocular deprivation). Of the 45 animals analyzed, 14 failed to reveal systematic activity at stimulation frequency (0.11 Hz) and were therefore not included in further analyses, leaving us with 31 animals (no manipulation: 8; Fluoxetine only: 6; EndoN only: 8; Fluoxetine and EndoN: 9). We found that animals not treated with Fluoxetine did not undergo any shift in ODI, regardless of whether they were intracerebrally injected with endoN (paired *t*-test before vs. after MD; *p* = 0.29), or with vehicle (paired *t*-test; *p* = 0.49), indicating that PSA removal did not influence basal visual responses. Fluoxetine treatment induced a significant shift of the ODI towards the non-occluded eye in the control (vehicle-injected) mice, consistent with previous findings in rats (Maya-Vetencourt et al., 2008; paired *t*-test; *p* = 0.035, Figure 3C). Similarly, Fluoxetine also produced a similar significant shift in the ODI in mice intracerebrally injected with endoN (paired *t*-test; *p* = 0.036, Figure 3C). This effect is also observed when comparing the shift of ODI with a 2-way ANOVA (factors: Fluoxetine and EndoN). We indeed found a significant effect of Fluoxetine (*F*_(1,27)_ = 5.102 and *p* = 0.032), no effect of endoN (*F*_(1,27)_ = 0.332 and *p* = 0.063), and no Fluoxetine-endoN interaction (*F*_(1,27)_ = 0.089 and *p* = 0.76). These results suggest that depletion of PSA-NCAM influenced neither the basal properties nor the fluoxetine-induced plasticity of the ocular dominance in the mouse visual cortex.

### Both Fluoxetine and EndoN Alter the Basal Activity of Parvalbumin Interneurons

**Figure 4:**
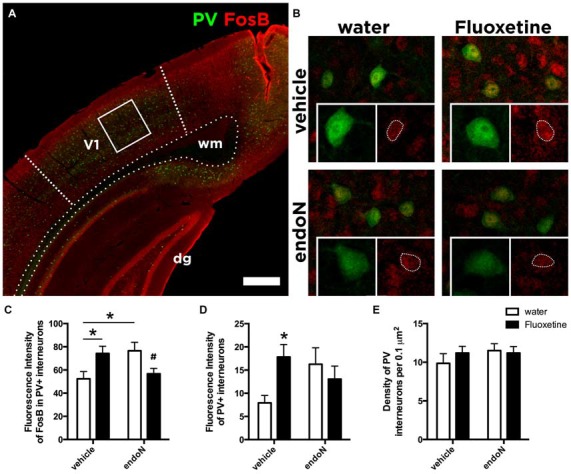
** Expression of FosB in Parvalbumin interneurons. (A)** Confocal single plane showing the expression of PV and FosB in the primary visual cortex of control mice. **(B)** Confocal single planes showing the expression of FosB in PV interneurons under different experimental conditions. Graphs showing the averages ± SEM of the intensity of fluorescence of **(C)** FosB in PV interneurons (water-vehicle = 52.38 ± 6.22, water-endoN = 76.58 ± 7.28, Fluoxetine-vehicle = 69.75 ± 7.20, Fluoxetine-endoN = 54.69 ± 4.7) and of **(D)** PV (water-vehicle = 7.93 ± 1.60, water-endoN = 16.25 ± 3.54, Fluoxetine-vehicle = 17.40 ± 2.93, Fluoxetine-endoN = 13.85 ± 2.99; 2-way ANOVA with Fisher’s LSD multiple comparison test; *p < 0.05; ^#^indicates that this group was not different from the control group but different from the other two groups; for more details, see “Results” section). **(E)** Graph showing the density of interneurons expressing PV per 0.1 μm in the primary visual cortex (water-vehicle = 9.86 ± 1.25, water-endoN = 11.50 ± 0.86, Fluoxetine-vehicle = 11.20 ± 0.86, Fluoxetine-endoN = 11.20 ± 0.83). Scale bar = 400 μm in **(A)** and 25 μm in **(B)**.

Parvalbumin (PV) expressing interneurons are considered key regulators of the critical period plasticity and their axon terminals are modulated by PSA-NCAM expression (Di Cristo et al., 2007; Castillo-Gómez et al., 2011). We hypothesize that PSA-NCAM modulation on the axon terminals might affect the synaptic activity of PV interneurons. We therefore analyzed the effects of chronic Fluoxetine treatment and intracerebral injection of endoN on the expression of the basal activity marker FosB (Nestler et al., 2001; Robison and Nestler, 2011) in PV interneurons in the primary visual cortex (Figures 4A,B). Fluoxetine treatment produced a significant increase of FosB expression in PV positive interneurons in animals injected with vehicle (Fisher’s LSD; *p* = 0.020; Figure 4C). In addition, endoN injection also increased FosB intensity in PV positive interneurons compared to control animals (Fisher’s LSD; *p* = 0.0146; Figure 4C). However, it is interesting to note that the combination of chronic Fluoxetine and the removal of PSA returned the expression of FosB to the basal control levels, indicating an interaction (2-way ANOVA; *F*_(1,31)_ = 11.36 and *p* = 0.0020).

Finally, we analyzed more in detail the expression of PV. By measuring the fluorescence intensity of PV, we found a trend in the interaction between both factors: endoN and Fluoxetine (2-way ANOVA; *F*_(1,31)_ = 3.908 and *p* = 0.0570; Figure 4D). In the multiple comparisons test, we found that in animals injected intracerebrally with vehicle, Fluoxetine treatment increased the fluorescence intensity of PV interneurons (Fisher’s LSD; *p* = 0.0362). However, the total number of neurons in the visual cortex was unaffected (2-way ANOVA; Fluoxetine: *F*_(1,31)_ = 0.2996 and *p* = 0.5881; endoN *F*_(1,31)_ = 0.7434 and *p* = 0.3952; Figure 4E).

## Discussion

In the present study, we have used ocular dominance plasticity in the mouse visual cortex as a model to investigate whether removal of PSA might influence the Fluoxetine-induced reactivation of critical period-like plasticity in the adult brain. We show that injection of the enzyme endoN produces a clear removal of PSA that after 2 weeks extends to the visual cortex and most parts of the telencephalon, indicating that the enzymatic treatment reliably disrupts the PSA side chains of NCAM in adult brain.

As previously reported in rats (Maya-Vetencourt et al., 2008), we found that chronic Fluoxetine treatment reopens a period of plasticity in the adult mouse visual cortex that resembles the original critical period of postnatal development. Specifically, chronic Fluoxetine treatment has also been demonstrated to produce an increase in PSA-NCAM expression and other molecules involved in inhibitory neurotransmission (Varea et al., 2007; Karpova et al., 2011; Ohira et al., 2013; Guirado et al., 2014a). On the other hand, removal of PSA by intracerebral infusion of endoN in the basolateral amygdala has been shown to enhance extinction of fear memories (Markram et al., 2007). At the cellular level, removal of PSA produces specific structural plasticity changes in different subpopulations of interneurons (Saarelainen et al., 2003; Varea et al., 2007; Guirado et al., 2014b).

Regarding visual plasticity, the decline of PSA-NCAM expression after early development has been linked to the earlier onset of critical period plasticity through an enhanced perisomatic innervation by PV interneurons in the visual cortex (Di Cristo et al., 2007). We hypothesized that the increased PSA-NCAM expression produced by Fluoxetine, resembling that high PSA-NCAM expression during development, might be necessary for the structural remodeling underlying the shifts in the ocular dominance. However, we have found that depletion of PSA by the injection of endoN influenced neither the basal ocular dominance conditions, nor the reactivation of juvenile-like plasticity induced by Fluoxetine. Therefore, our results suggest that PSA-NCAM is not required for the plasticity triggered by Fluoxetine in the visual cortex.

The opening and closure of critical periods in the visual cortex has been suggested to be dependent on the remodeling of inhibitory circuits (Yazaki-Sugiyama et al., 2009; Harauzov et al., 2010), and therefore the disruption the excitatory/inhibitory balance (Hensch, 2005). Specifically, modulation of PV interneurons activity has been suggested to play a role in triggering critical period plasticity (Hensch, 2005; Kuhlman et al., 2013).

Therefore we decided to study the expression of FosB as a marker of basal long-term activity (Nestler et al., 2001; Robison and Nestler, 2011; Nestler, 2015) in PV interneurons. While we have found that Fluoxetine increases the expression of PV in control animals, we also show that both chronic Fluoxetine treatment and PSA removal, independently produce an increase in the expression of FosB in PV interneurons. This is in line with a very recent study that showed an increased FosB expression throughout the brain after Fluoxetine treatment (Vialou et al., 2015). It is also in agreement with a previous study that indicated an increase in the density of PV and PV/synaptophysin expressing puncta on the perisomatic region of pyramidal neurons after PSA removal (Castillo-Gómez et al., 2011), what suggests an increased activity of PV interneurons. In fact, PSA removal has previously been suggested to lead to increased activity via BDNF (Burgess and Aubert, 2006). However, it is interesting to note the negative interaction between both factors as well, suggesting they act through different mechanisms on PV interneurons. Further experiments including control animals in which no monocular deprivation is carried out, might help to understand this negative interaction.

Since we observe an increase in the FosB expression in PV interneurons in animals only injected with endoN, these results indicate that the average changes of activity of PV interneurons are not sufficient to trigger adult brain plasticity. As it has been suggested previously, brain plasticity might depend on the activity of few selected PV hub interneurons (Bonifazi et al., 2009; Buzsáki, 2010; Royer et al., 2012; Cossart, 2014). Therefore, it might be that our study masks the specific changes in the activity of those few selected hub PV interneurons. Nevertheless, further studies will be necessary to understand the mechanisms through which antidepressants, such as Fluoxetine, trigger critical period plasticity in the adult brain.

## Author Contributions

RG, JU, PS and EC designed research; RG, DLT and HC performed research; RG and MB analyzed data; JN, MB, RG and EC wrote the article.

## Funding

This study was supported by ERC grant No 322742-iPLASTICITY, Sigrid Juséliuksen Säätiö foundation and Academy of Finland grant #257486.

## Conflict of Interest Statement

The authors declare that the research was conducted in the absence of any commercial or financial relationships that could be construed as a potential conflict of interest. EC is an advisor and shareholder in Herantis Pharma, Inc.
